# Pembrolizumab induced remission of recurrent and metastatic sinonasal squamous cell carcinoma after overcoming checkpoint‐inhibitor pneumonitis: A case report and literature review

**DOI:** 10.1002/cnr2.1778

**Published:** 2023-01-05

**Authors:** Deepak Rajendran Nair, Ram Trehan

**Affiliations:** ^1^ Medical Oncology & Hematology Greater Washington Oncology Associates Silver Spring Maryland USA

**Keywords:** checkpoint‐inhibitor pneumonitis, immunotherapy, pembrolizumab, sinonasal squamous cell carcinoma

## Abstract

**Background:**

For programmed death‐ligand‐1 (PD‐L1) positive recurrent and metastatic head and neck squamous cell carcinoma (R/M‐HNSCC), KEYNOTE‐048 and KEYNOTE‐040 clinical trials recently approved pembrolizumab monotherapy as first‐line treatment. However, recurrent and metastatic sinonasal squamous cell carcinoma (R/M‐SNSCC) was excluded from these clinical trials and treatment reports of immune‐checkpoint inhibitor (ICI) in R/M‐SNSCC are sparse. Immune‐related adverse events (irAEs) are known to occur during ICI treatment and some of these such as checkpoint‐inhibitor pneumonitis (CIP) can be fatal. ICI rechallenge after severe irAEs is debated.

**Case:**

We describe a case of a 65‐year‐old male with R/M‐SNSCC who is currently in remission with pembrolizumab monotherapy. He developed high‐grade pneumonitis during the course of treatment warranting ICI discontinuation but has since tolerated full‐dose pembrolizumab for 10 months now which is holding his disease stable. Our approach toward restarting full‐dose pembrolizumab was by monitoring the patient's response to an initial low dose of pembrolizumab with concomitant oral steroid immunosuppression to control CIP.

**Conclusion:**

Clinicians should weigh the risk‐to‐reward ratio of ICI rechallenge after improvement of high‐grade CIP, particularly for selected patients with aggressive tumors such as R/M‐SNSCC and prior treatment response. Under close monitoring, ICI resumption at a low dose and assessing patient tolerance with concomitant immunosuppression may be a reasonable approach to reintroducing ICI after high‐grade CIP in these patients.

## 1 BACKGROUND

Sinonasal squamous cell carcinoma (SNSCC) is an uncommon but aggressive type of head and neck squamous cell carcinoma (HNSCC) affecting fewer than 1 person per 100 000 individuals in the United States.^1^ In contrast to oral and laryngeal cancers which constitute the majority of HNSCC, symptoms due to the primary SNSCC tumor are frequently innocuous. This often results in diagnosis once the tumor is advanced and poorer survival rates.^1^, ^2^ The therapeutic landscape of recurrent and metastatic (R/M) HNSCC has recently been revolutionized following the discovery of their programmed death‐ligand‐1 (PD‐L1) expression which are targets for immune‐checkpoint inhibitor (ICI) therapy.^3^, ^4^ However, R/M‐SNSCC were excluded from these key trials exploring ICI despite their similar immunogenicity.^5^, ^6^

Pembrolizumab is an anti‐PD1 class of ICI that enhances the body's immune response to kill tumor cells. However, the same pathway that is responsible for the efficacy of the treatment also causes immune‐related adverse effects (irAEs) which are excessive reactions to normal cells due to reinvigoration of the immune system.^7^ Checkpoint‐inhibitor pneumonitis (CIP) is an uncommon but potentially life‐threatening irAE.^7^, ^8^ Current literature has mixed evidence regarding ICI rechallenge after improvement of irAEs.^9^, ^10^, ^11^ Moreover, reports of ICI treatment, irAE incidence, and ICI rechallenge in HNSCC, particularly in SNSCC, are sparse. This case report describes a case of R/M‐SNSCC that is currently under remission following successful ICI rechallenge after overcoming CIP.

## 2 CASE PRESENTATION

**FIGURE 1:**
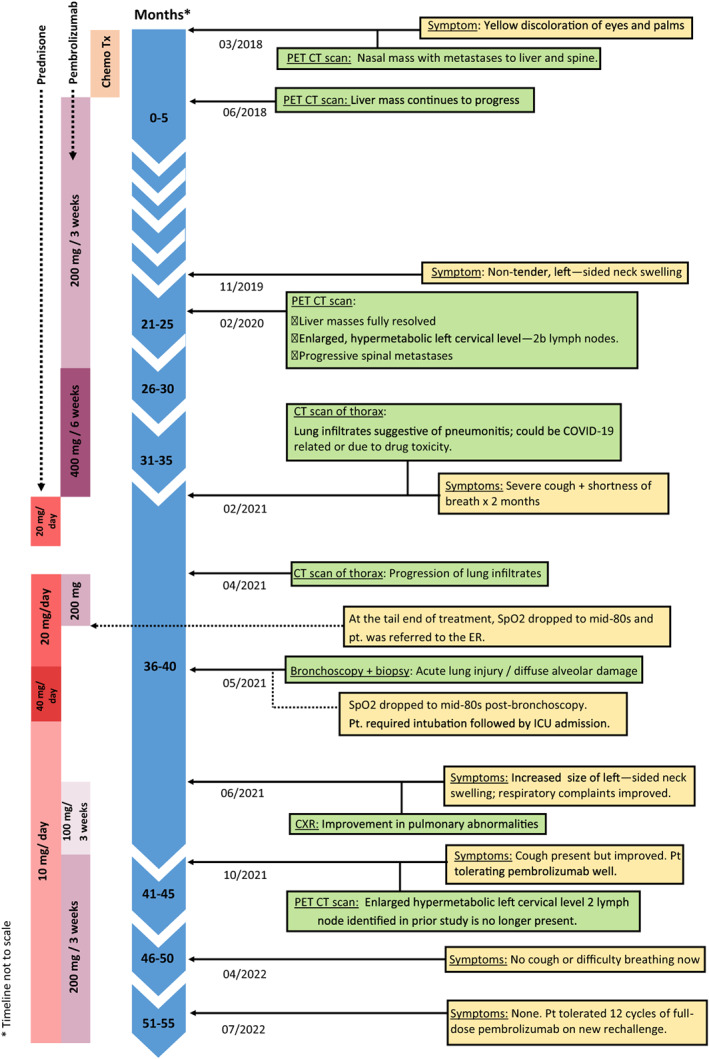
Timeline of key events.

In March 2018, a 65‐year‐old male presented to the outpatient department for evaluation of yellow discoloration of eyes and palms for 3‐month duration (Figure 1). PET‐CT scan revealed a liver mass measuring 1.5 cm, a mass in the left nasal cavity, and spinal metastases. Biopsy revealed squamous cell carcinoma in the posterior nasal cavity and a liver biopsy confirmed the same pathology. He was initially started on 5‐FU, carboplatin, and cetuximab, however, the liver mass continued to progress. The patient was started on pembrolizumab monotherapy 200 mg every 3 weeks after results of 95% PD‐L1 positivity.

**FIGURE 2:**
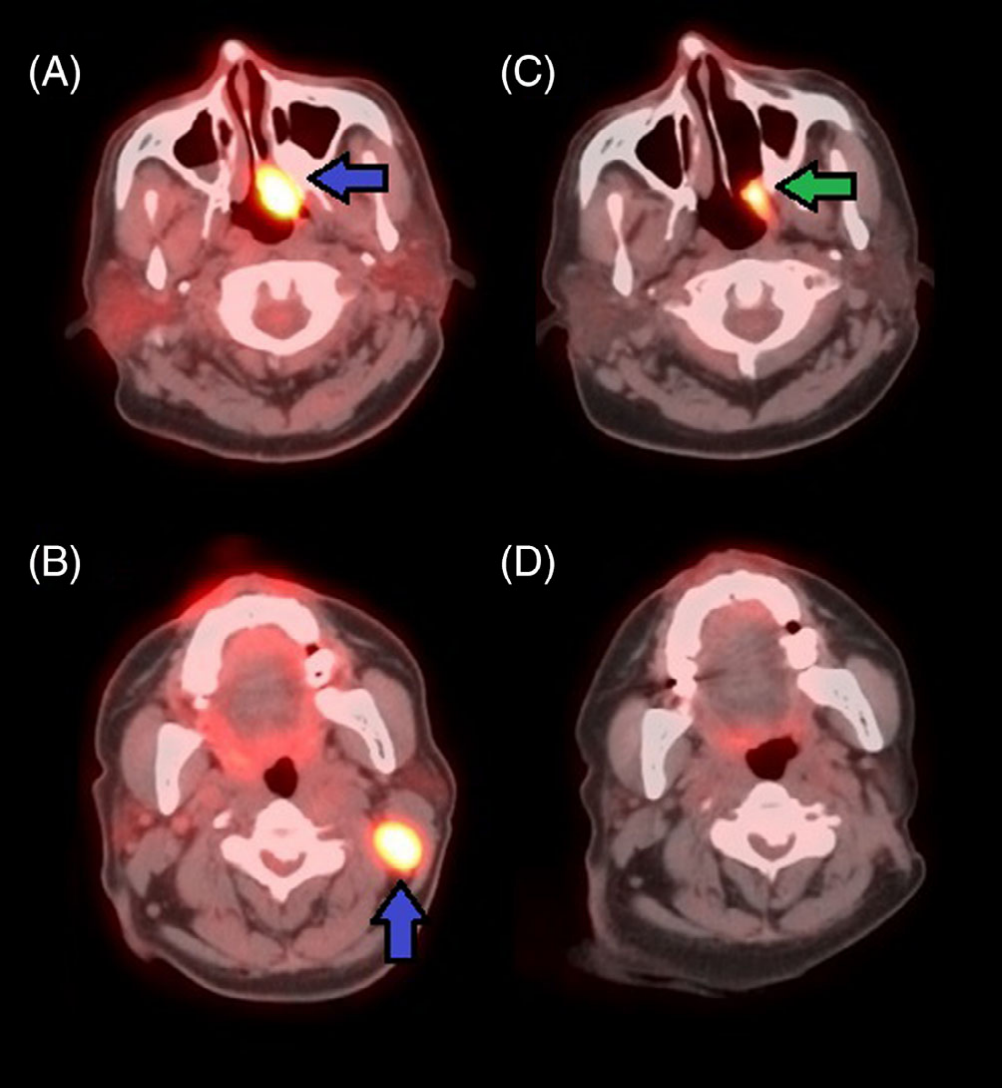
(A, B) PET/CT scan in February 2020: Hypermetabolic left posterior nasal cavity mass consistent with patient's known malignant tumor measuring 1.5 × 2.7 cm demonstrating a maximum SUV of 16.2 (blue, left arrow). Enlarged hypermetabolic left cervical level IIb lymph node consistent with regional metastasis measuring 1.8 × 2.2 cm with a maximum SUV of 15.6 (blue, up arrow). (C, D) PET/CT scan in October 2021: Hypermetabolic triangular shaped mass in the left posterior sidewall of the nasopharynx that appears to be decreased in size when compared to prior study; mass measures 1.1 × 2.2 cm with a maximum SUV of 8.9 (green, left arrow). Enlarged hypermetabolic left cervical level IIb lymph node identified on previous study is no longer present.

After 14 cycles of pembrolizumab, his PET‐CT scan in February 2020 revealed complete resolution of the liver mass. However, there was a new enlarged hypermetabolic left cervical level IIb lymph node (Figure 2) and progressive spinal metastases. Between June 2020 and February 2021, the dose of pembrolizumab was increased to 400 mg every 6 weeks to control disease progression. At his visit in February 2021 after six cycles on the new infusion dose, he complained of severe cough and shortness of breath of 2 months duration. CT scan of the chest revealed features of organizing pneumonia consistent with pneumonitis. Test for COVID‐19 and sputum cultures were negative. PFT revealed moderate restriction with an FEV1/FVC 80%; FVC 2.25 (66% predicted); FEV1 1.80 (70% predicted); TLC 2.97 (53% predicted); RV 0.73 (37% predicted); DLCO 15.2 (15.9 predicted); DLCO/VA 4.69 (97% predicted). As similar findings could also be seen with pneumonitis due to drug toxicity, the patient was advised to begin oral prednisone 20 mg daily and to defer pembrolizumab initially for 4 weeks while monitoring response to steroids.

**FIGURE 3:**
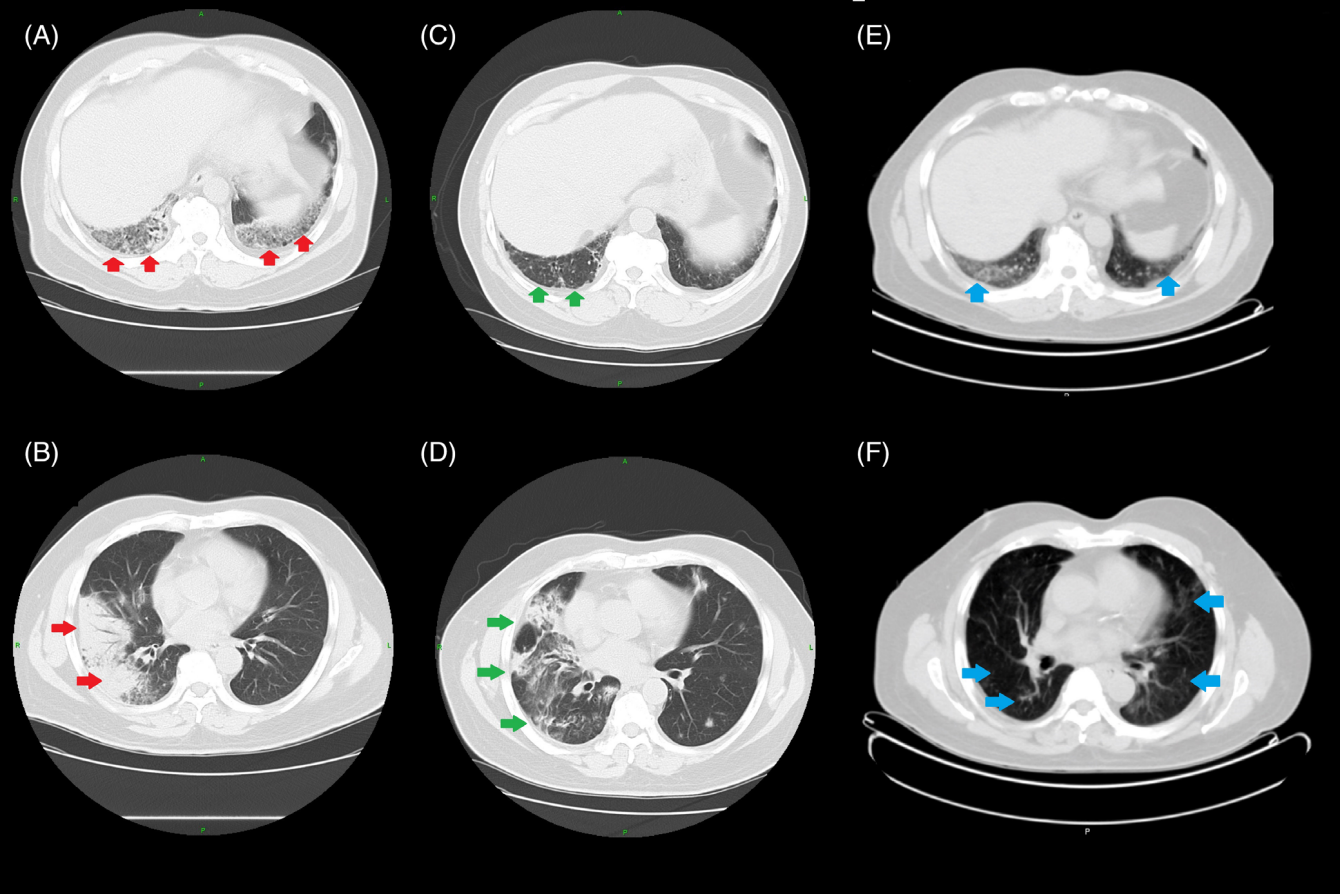
(A, B) CT scan in February 2021: Focal consolidative airspace disease in the posterior lateral right lower lobe and posterior basilar left lower lobe. Multifocal, peripheral ground‐glass opacities with round morphology more numerous in the right lung. Features are consistent with organizing pneumonia seen with drug toxicity. (C, D) CT scan in April 2021: Improved dense consolidation in the right lower lobe but the multiple small areas of focal consolidation within the right upper lobe on the previous examination have progressed since the previous study and there are also multiple new areas of focal irregular consolidation with central air bronchograms involving both lungs representing inflammatory process. (E, F) CT scan in October 2021: Diffuse ground‐glass infiltrates bilaterally in the lungs demonstrating mild metabolic activity with SUV of 2.2 concerning for unresolved pneumonitis.

Four weeks later, in March 2021, the patient reported improvement in his cough and shortness of breath and was, hence, taken off steroids. However, the annual restaging PET/CT scan done 1 month later revealed the progression of organizing pneumonia of both lungs compared to the previous CT scan done in February 2021 (Figure 3). Re‐evaluation for COVID‐19 and repeat sputum cultures were negative. Based on these imaging results, a trial of oral prednisone 20 mg daily for 2 weeks was advised while the patient awaited bronchoscopy to evaluate for recurrent cancer. At this juncture, it was decided to proceed with pembrolizumab since his risk of downward clinical course was higher with metastatic cancer compared to drug toxicity. This decision was based on the clinical improvement of the patient's pulmonary symptoms, our doubts if recurrent cancer was the cause of progressive lung findings in the recent CT scan, and prior response to pembrolizumab which had resulted in partial remission. Pembrolizumab was challenged at half‐dose 200 mg at this office visit, however, the patient developed hypoxia at the tail end of treatment (SpO_2_ dropped to the mid‐80s) and had to be rushed to the emergency room.

A week later, the patient underwent bronchoscopy evaluation for lung biopsy. There was no evidence of infectious etiology, metastases or flow immunophenotypic evidence of lymphoproliferative disorder, however, the lung biopsy revealed organizing pneumonia with features of underlying acute lung injury along with patchy fibrosis and honeycombing suggestive of chronic lung disease. Post‐bronchoscopy, the patient's SpO_2_ dropped to the mid‐80s and required intubation followed by ICU admission. Following extubation, his oral prednisone dose was increased to 40 mg daily for 3 weeks.

In May 2021, 3 weeks after the above events, it was decided to pause pembrolizumab until the patient's lung performance had improved and to continue oral steroids with an appropriate tapering dose till that time. The patient understood that while this would put him at risk of tumor progression, the decision had to be weighed against the risk of repeated insult to his compromised lungs with the immunotherapy.

**FIGURE 4:**
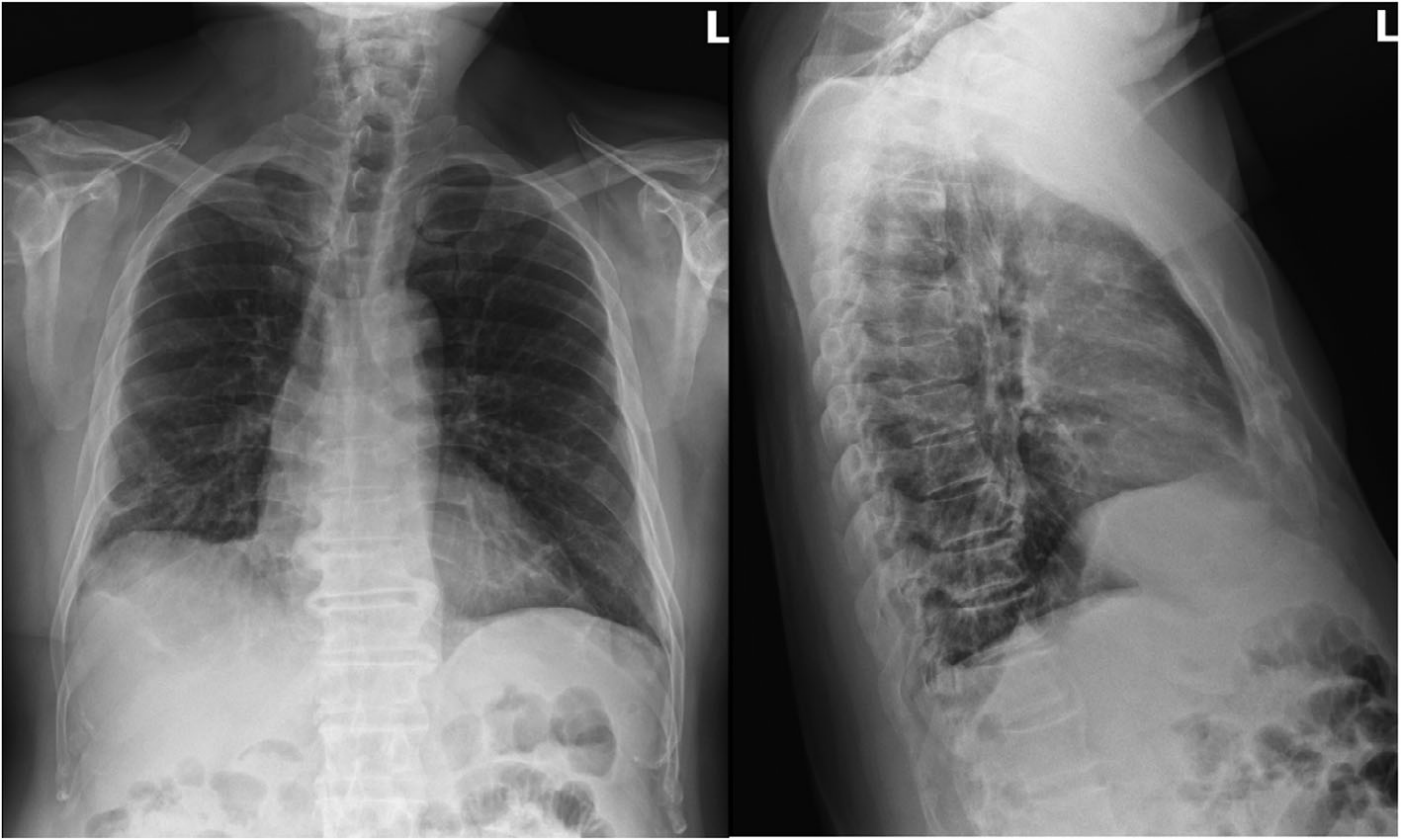
Chest X‐ray in June 2021: Improved aeration of the lungs with decreasing peripheral based opacities, compared to CT chest done in April 2021, most notably in the right lung. No new focal consolidation, effusion, or pneumothorax.

Four weeks later, in June 2021, the patient reported improvement in his respiratory symptoms and a chest X‐ray done at that time reflected the same (Figure 4). However, he reported an increase in the size of left‐sided neck swelling suspicious of tumor progression as he was off pembrolizumab for 6 weeks since the last immunotherapy challenge and for 8 weeks prior to that. The patient was resumed on pembrolizumab 100 mg every 3 weeks which he began to tolerate well. It was decided to continue the current regimen and gradually increase the dose based on the patient's response.

Annual PET/CT restaging in October 2021 revealed complete resolution of the enlarged hypermetabolic left cervical level IIb lymph node identified in the prior study done in February 2020 (20 months ago) (Figure 2). However, there were diffuse ground‐glass infiltrates bilaterally in the lungs suggesting persistent pneumonitis. The patient still had trouble getting off oral steroids: cough would worsen whenever the dose was reduced below 10 mg. As the patient had tolerated five cycles of three weekly 100 mg pembrolizumab well, it was attempted to increase the dose of pembrolizumab to 200 mg every 3 weeks.

As of July 2022, our patient has tolerated 12 cycles of full‐dose pembrolizumab. His respiratory symptoms are concurrently being treated with 10 mg oral prednisone daily by the pulmonology team. He has reported no cough or shortness of breath since April 2022. PET/CT scan are being planned for future follow‐ups.

## 3 DISCUSSION

The anti‐PD1 class of ICI therapy has established itself as a frontier of treatment of PD‐L1 positive R/M‐HNSCC based on the recent KEYNOTE‐040 and KEYNOTE‐048 clinical trials. Current evidence for the treatment of R/M‐SNSCC with anti‐PD1 is based on the regimen approved for the treatment of R/M‐HNSCC as the former were excluded from these clinical trials.^3^, ^4^ PD‐L1 expression in R/M‐SNSCC offers a new treatment strategy and hope in the outlook of these aggressive tumors with otherwise limited treatment options and poor prognosis.^6^, ^12^

The incidence of CIP in the patient group receiving pembrolizumab monotherapy was 2.4% and less than 1% in KEYNOTE‐040 and KEYNOTE‐048 respectively.^3^, ^4^ Although rare in incidence, CIP can be fatal and accounts for 35% of PD‐1 and PD‐L1 inhibitor‐related deaths.^7^ Clinicians should suspect CIP at its earliest reported symptoms: the most common symptoms are dyspnea and cough while fever and chest pain are less frequently reported.^8^, ^13^ The onset of these symptoms during ICI therapy warrants a detailed evaluation to assess disease progression, CIP, or incidental events related to cancer or prior medical conditions.^14^ ICI interruption coupled with immunosuppression with steroids is the cornerstone of treatment for CIP.^13^, ^15^, ^16^

Current guidelines on ICI rechallenge after irAE are based on clinical observations and expert consensus but not prospective clinical trials.^13^, ^15^, ^16^ Our patient initially had Grade 2 CIP warranting temporary ICI discontinuation but developed severe CIP (Grade 3–4) upon the first ICI rechallenge with 200 mg pembrolizumab. Although the current recommendation is permanent discontinuation of ICI therapy following severe CIP, we decided to begin a second rechallenge by closely monitoring patient tolerance to an initial trial of 100 mg pembrolizumab every 3 weeks with concurrent oral steroid immunosuppression and gradually reintroducing full‐dose 200 mg pembrolizumab. This decision was made after discussing the benefit‐to‐risk ratio with our patient who reported symptoms suggestive of cancer progression during the period of ICI interruption as well as his prior treatment response to anti‐PD1 and very high tumor PD‐L1 expression.

Previous studies have suggested that ICI rechallenge may be done with caution following severe irAE.^11^, ^17^ Haanen et al have suggested that single‐agent rechallenge with concurrent immunosuppression after high‐grade irAE may be done in select patients with well‐controlled initial irAE if the oncologic situation requires it.^11^ We did not consider rechallenging with an alternate class of ICI as the patient had shown excellent prior response to pembrolizumab and given the high tumor PD‐L1 expression.

It is possible that our patient is treated at significant risk and is fortunate to have cancer under control as a result. However, R/M‐SNSCC is an aggressive tumor with limited treatment options. We would like to reemphasize that the decision to rechallenge pembrolizumab was made after careful consultation with the patient who fully understood the risks of CIP recurrence. Further, the volume and dosing schedule of pembrolizumab were modified from the standard due to concerns about irAE recurrence. As is the case with our patient, the ICI rechallenge should be decided only after evaluating its benefits and risks with the patient. Our patient is being closely monitored for any adverse effects.

There is emerging literature suggesting that irAE development is significantly associated with response to ICI.^18^, ^19^, ^20^ Our patient achieved remission despite ICI hold to allow recovery from CIP. It is possible that CIP development may have heralded clinical response in our patient, however, guarantee‐time bias is a potential confounding with this association as patients who are on ICI for longer periods tend to develop irAEs and are hence more likely to respond to treatment.^21^

## 4 CONCLUSION

We present this case to discuss the treatment outcomes of anti‐PD1 in PD‐L1 positive R/M‐SNSCC and to emphasize the importance of early diagnosis and management of CIP. Clinicians should weigh the risk‐to‐reward ratio of ICI rechallenge after improvement of high‐grade CIP, particularly for selected patients with aggressive tumors such as R/M‐SNSCC and prior treatment response. Under close monitoring, ICI resumption at a low dose and assessing patient tolerance with concomitant immunosuppression may be a reasonable approach to reintroducing ICI after high‐grade CIP in these patients. Further work is needed in this area to dictate practice algorithms.

## AUTHOR CONTRIBUTIONS

**Deepak Rajendran Nair:** Conceptualization (equal); data curation (lead); visualization (lead); writing – original draft (lead); writing – review and editing (equal). **Ram Trehan:** Conceptualization (equal); supervision (lead); writing – review and editing (equal).

## CONFLICT OF INTEREST

The authors declare that they have no known competing financial interests or personal relationships that could have appeared to influence the work reported in this paper.

## PATIENT CONSENT

The authors declare that they have obtained written consent from the patient.

## Data Availability

Data available on request due to privacy/ethical restrictions.
